# Scenting serenity: influence of essential-oil vaporization on dental anxiety - a cluster-randomized, controlled, single-blinded study (*AROMA_dent*)

**DOI:** 10.1038/s41598-024-63657-w

**Published:** 2024-06-19

**Authors:** Judith Czakert, Farid I. Kandil, Hiba Boujnah, Pantea Tavakolian, Sarah B. Blakeslee, Wiebke Stritter, Henrik Dommisch, Georg Seifert

**Affiliations:** 1 Department of Pediatric Oncology and Hematology, Research Group: Prevention, Integrative Medicine and Health Promotion, Charité – Universitätsmedizin Berlin, Corporate Member of Freie Universität Berlin and Humboldt Universität zu Berlin, Berlin, Germany; 2 Department of Nephrology and Medical Intensive Care, Charité – Universitätsmedizin Berlin, Corporate Member of Freie Universität Berlin and Humboldt Universität zu Berlin, Berlin, Germany; 3 Institute of Social Medicine, Epidemiology and Health Economics, Charité – Universitätsmedizin Berlin, Corporate Member of Freie Universität Berlin and Humboldt Universität zu Berlin, Berlin, Germany; 4 Department of Periodontology, Oral Medicine and Oral Surgery, Dentistry, Charité – Universitätsmedizin Berlin, Corporate Member of Freie Universität Berlin and Humboldt Universität zu Berlin, Berlin, Germany

**Keywords:** Dental fear and anxiety (DFA), Aromatherapy, Essential oil, Complementary and integrative medicine (CIM), Prevention, Health care, Health occupations, Signs and symptoms

## Abstract

Dental fear and anxiety (DFA) is known as an immense challenge in oral healthcare, which can result in compromised oral health, pain, and uncomfortable treatment. The objective of this study was to analyze the effect of essential-oil vaporization on acute anxiety of patients in dental practices. Four dental practices used five weekly cycles of vaporization with each scent: Orange (Citrus sinensis), Swiss Pine (Pinus cembra), Good Mood (blended essential oils: Citrus sinensis, Citrus aurantifolia, Citrus limon, Osmanthus fragrance (5%)), Forest Walk (blended essential oils: Abies grandis, Pinus cembra, Myrtus communis c. t. 1,8-cineol, Abies alba, Citrus paradisi, Abies sibirica, Pseudotsuga menziesii, Vetiveria zizanoides), and water. Acute anxiety was the primary outcome (state-trait-anxiety inventory (STAI-S)). Secondary outcomes were trait anxiety (STAI-T), dental anxiety (Kleinknecht dental fear survey), and pain perception in treatment (numeric rating scale). Across all patients (n = 486), STAI-S was slightly higher in the control group (40.7 ± 11.6) than in the intervention groups (38.4 ± 10.5). Post-hoc analyses revealed that the effect is only robust for the subgroup of female patients (n = 296, *p* = 0.044). We also conducted a post-hoc additional analysis on a subpopulation with an increased level of STAI-T ≥ 42 (n = 131 patients). For this group the difference in acute anxiety between the control group (51.1 ± 11.9, n = 30) vs. the intervention groups (46.8 ± 9.6, n = 118) was significant (*T* = 4.39, *p* = 0.0379). The results of the study indicate a promising potential of essential-oil vaporization to alleviate dental anxiety, particularly in the subgroups of patients with a high level of trait anxiety, and particularly in female patients. The calming effects of the essential-oil vaporization were also highlighted by the anecdotical statements of the dental-practice staff. The anxiety-reducing role of essential-oil vaporization alone and as one part of combined techniques to counter DFA should be further explored using multi-perspective methodological approaches in research.

## Introduction

Dental anxiety has been a topic of scientific attention since the 1970’s^1^. The term refers to the specific reaction of patients to dental treatment-related stress^2^. Dental fear, however, is explained by the feeling that arises in connection with specifically occurring impulses^3^, for instance the sound of drills or the smell of dental practices. Dental fear and anxiety (DFA) create challenges in oral healthcare not only for patients’ well-being and health but also for the dental care team^4,5^. The global prevalence of DFA is estimated at 15.3% with any DFA, 12.4% with high DFA, and 3.3% with severe DFA whereby mostly women seem to be affected^3^. Treating patients with DFA may result in increased time required for treatment or a stressful experience for both patients and dentists due to the anxiety and corresponding reactions^5^. Moreover, research indicates that DFA increases the pain perception of patients during treatment^6^. The negative effects of DFA might also intensify in a vicious cycle of anxiety^7,8^. Hence, dealing with DFA is considered a challenging task for dentists and dental staff^9^.

Based on these findings, the need for strategies to prevent and treat DFA is apparent and currently reflected in several approaches: pharmacological management of patients with a high level of DFA is well established^10^. Also, non-pharmacological techniques are frequently used, such as distraction, relaxation, providing information about the treatment and establishing a trusting relationship^7^. This also includes methods used in complementary and integrative medicine (CIM) to reduce stress and anxiety, and enhance general well-being, such as progressive muscle relaxation, guided imagery, hypnosis, music therapy, and acupuncture^5,7,11^. The specific use of stress-reducing essential oils via inhalation, also known as aromatherapy, is also considered to be effective at reducing anxiety and pain in health related research^12^ including pain experienced during dental care^5–7,11,13^. Overall, applying aromatherapy with essential oils has the advantage of potentially being beneficial to all dental patients, regardless of their DFA level.

### Aromatherapy

Essential oils are “mixture[s] of highly reactive, volatile, mostly fragrant chemical compounds”^14^ (quote translated by JC) extracted from plant components such as flowers, peels, resins, wood, bark, and roots. Under the term aromatherapy, essential oils are used mostly via inhalation and/or percutaneously^15^ for health promotion and disease relief as a supportive treatment approach in health related contexts^16^. The complex composition and the multitude of ingredients of essential oils are considered the basis for the potential of aromatherapy for health and well-being. The synergistic effects are assumed to go beyond the effects of the individual ingredients^15,17,18^. In addition, there are indications that a blend of different essential oils could enhance positive effects further^19^ while minimizing risks^14,20^.

According to current knowledge, the effects of essential oils are explained by two different modes of action/principles: psychological and pharmacological^21,22^. The psychological principle refers to individual and culturally shaped experiences associated with an odor that lead to subjective reactions. Thus, the same essential oil can trigger completely different reactions in different people. The pharmacological mechanism, on the other hand, is based on the specific composition of the essential oils and the affinity of its components to certain receptors. This is accompanied by a specific dose–response relationship and a substance specificity that is independent of cognitive control mechanisms. It can be assumed that in aromatherapy allocated through inhalation (e.g., via room vaporization), the psychological mechanisms of action predominate^22,23^. Accordingly, the explanations of the biological mechanism underlying the specific anxiolytic effect of essential oils have not been conclusively clarified^12^. Explanatory models invoke, for example, the influence of essential oil components on neurotrophic factors, the endocrine system, and neurogenesis^19^. Another assumption relates to the hypothesis that a subjectively positive association with odors could have a positive influence on emotions and thus an alleviating effect on acute anxiety^22,24^.

Despite the growing conviction that aromatherapy has the potential to reduce DFA, and pain, and to enhance the well-being of dental patients^5^, evidence underscoring these effects is still limited^11^. Some reasons can be related to the specific characteristics of the research field: Comparability is hampered because of differences in, for instance, essential oils used, context of application, and sample size. Moreover, transparency regarding the information about the essential oils used (e.g., manufacture, botanical names of the ingredients, composition), the devices for vaporizing, and the contextual conditions of the essential oil application (e.g., odor intensity, characteristics of the premises) is not always provided^23,25^. Furthermore, although the number of reviews on aromatherapy as an intervention against DFA^5–7,11,13^ suggests growing research activity on the topic, research gaps can be identified. For instance, no research about aromatherapy limiting DFA has considered the relevance of individual reactions on smell, and in all studies, only singular essential oils and no essential oil blends were used to our knowledge.

### Research objective and design-shaping context

Subsequently, this study aimed to investigate the efficacy of aromatherapy on the acute state of anxiety and pain at the dentist, considering the psychological mechanism of action in terms of culturally shaped olfactory experiences in the study design.

Taking the psychological mechanism of action of essential oils into account, especially regarding the association of memories and cultural imprints with odor, essential oils used should meet two requirements. (1) They should cause physical relaxation given their pharmacological properties, and (2) they should be associated with relaxation and well-being by the greatest possible number of people to create a similar psychological effect. In many different cultures, the scent of forests is associated with relaxation, positive connotations, and memories. Associations with forests often coincide with idealized concepts of peacefulness, stillness, and closeness to nature, and are frequently closely linked to positive emotions and moods. This corresponds to the positive effect of the forest on the body and mind as shown by the multitude of scientific reviews on forest bathing^26–29^, forest therapy^30^, and nature therapy^31^. Nature-therapeutic approaches are also receiving increasing attention in the public presentation as health-promoting, preventing concepts.

Accordingly, and in line with the psychological effects of olfactory stimuli, it can be hypothesized that the scent of forest is associated with relaxation and may therefore have stress and anxiety-reducing effects in many people. Given this background, four different essential oils resp. oil blends were selected after consultation of professional aromatherapists from the company Primavera® for investigation regarding their anxiety-relieving effects in the dentistry setting: “Orange”, “Zirbelkiefer” (Swiss Pine), “Waldspaziergang” (Forest Walk), and “Gute Laune” (Good Mood). Rationales for the selection of essential oils and detailed information about their characteristics—orientated on the TREATS checklist (transparent reporting for essential oil & aroma therapeutic studies)^32^—are summarized in Table 1.

**Table 1:** Essential-oil characteristics.

Essential oils (batch number)	Ingredients/gas chromatography	Plant part/production method/cultivation method/country of origin^a^	Selection rationale	External references for selection rationale
Orange (00051K30)	Sweet Orange (Citrus sinensis) Approx. 95% limonene, in small amounts beta-myrcene, alpha-pinene and sabinene	**Citrus sinensis** peel/cold-pressing/organic cultivation/Italy, Spain, Mexico	Effect demonstrated in empirical research on dental anxiety. The effect of the essential oil is described as anxiety relieving. > Hypothesis 1	^33–38^
Gute Laune, Good Mood (00379K30)	Sweet Orange, Lime, Lemon, Litsea, Osmanthus Absolue 5% (Citrus sinensis 25–50%, Citrus surantifolia 10–25%, Citrus limon 10–25%, Litsea cubeba 10–25%, Osmanthus Fragrance (5%) 1–5%) Approx. 67% limonene, also citral, beta-pinene and gamma-terpinene	**Citrus sinensis** peel/ old-pressing/organic cultivation/Italy, Spain, Mexico **Citrus aurantifolia** peel/cold-pressing/organic cultivation/Brazil, Mexico **Citrus limon** peel/cold-pressing/organic cultivation/Italy, Argentinia	Essential oil blend based on orange and other citrus fruits to check if the mixture is more potent than the corresponding mono oil Orange, concerning the anxiolytic effect. > Hypothesis 1, 2	^14,20,39^
Zirbelkiefer, Swiss Pine (00186L30)	Swiss Pine (Pinus Cembra) Approx. 44% alpha-pinene, also beta-phellandrene, limonene and beta-pinene	**Pinus cembra** branches/distillation/organic cultivation/Austria, Italy	Swiss Pine is associated with the smell of coniferous forests. > Hypothesis 3 The effect of the essential oil is described as relaxing, anxiety-relieving, calming. > Hypothesis 1	^17,40–43^
Waldspaziergang, Forest Walk (00564J30)	Giant fir, Swiss Pine, Turkish myrtle, silver fir, grapefruit, spruce needle, Douglas fir, vetiver (Abies grandis 10–25%, Pinus cembra 10–25%, Myrtus communis c. t. 1,8-cineol 1–5%, Abies alba 1–5%, Citrus paradisi 10–25%, Abies sibirica 10–25%, Pseudotsuga menziesii 10–25%, Vetiveria zizanoides 1–5%) Approx. 33% limonene, also alpha-pinene, beta-pinene and beta-phellandrene	**Pinus cembra** branches/distillation/organic cultivation/Austria, Italy **Abies grandis** branches/distillation/organic cultivation/France **Abies sibirica** branches/distillation/collection of wild plants/Russia **Vetiveria zizanoides** roots/distillation/organic cultivation/Haiti, Madagascar	Essential oil blend with Swiss Pine and Orange and other essential oils, associated with forests and for stress reduction, to check if the mixture is more potent than the corresponding mono oil Swiss Pine and Orange, concerning the anxiolytic effect. > Hypothesis 1, 2, and 3	^14,20,42–44^

^a^In the blended essential oils (Forest Walk, Good Mood), the essential oils specified by the company as the main ingredients are described here.

Based on the outlined considerations, the study investigated the following hypotheses:

- 1. Aromatherapy with stress reducing essential oils evaporated in dental practices has an alleviating effect on patients' feelings of acute anxiety.
- 2. The anxiety reducing effect of the essential-oil blends (Good Mood and Forest Walk) is stronger than that of the corresponding mono oils (Orange and Swiss Pine).
- 3. The forest associated essential oil (Swiss Pine) and the corresponding blend (Forest Walk) show the strongest effect compared to the essential oil Orange and the corresponding blend Good Mood.
- 4. The anxiolytic effect of the essential oil vaporization corresponds to a lower subjective pain perception in patients during the treatment.

### Material and methods

A controlled, single-blinded, cluster-randomized design with four dental practices in Berlin was conducted between October and December 2022. The study was registered in the German Clinical Trials Register (DRKS00027233, 16/11/2021*)* and followed the CONSORT reporting guidelines^45^.

### Study design

**Figure 1:**
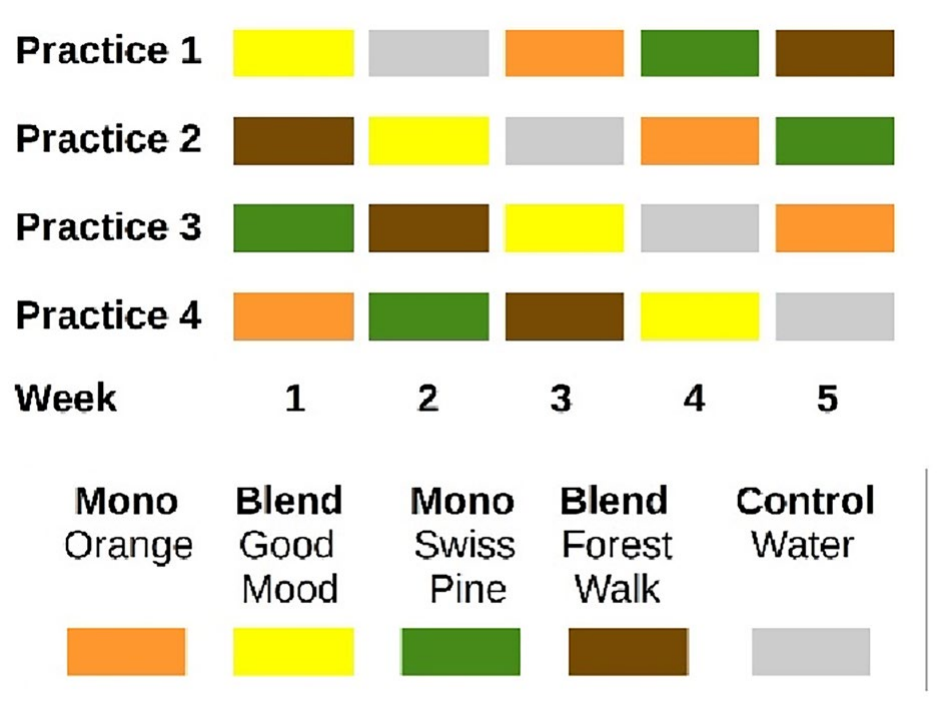
Study design.

In the selection process of dental practices, care was taken to ensure a wide variation in size, practice layout, location, and patient clientele in order to target and collect data on a broad group sample. Each of the five conditions (Orange, Swiss Pine, Forest Walk, Good Mood, and water as a control, cf. Figure 1 and Table 1) was tested in each practice for 1 week for data collection. The order of five conditions was put into a random order and each practice started with a different condition also randomized (using randomization routines provided in Python). This ensured a balanced design wherein each week, each practice utilized a different essential oil. (cf. Figure 1). In that way, patients were allocated to their intervention group based on the week of their appointment. Data collection took place in all four practices for a total of 5 weeks between October 10th and November 11th, 2022. However, it was extended by an additional 5 weeks in practice 1 and by 1 week in practice 3 in order to reach the calculated sample size.

Essential oil vaporization was performed in both the waiting areas and treatment rooms. For the waiting rooms, one large app-controlled vaporizer (HAAL ROSA) was used in each practice. Settings were adjusted to a medium diffusion intensity that was adapted to size and layout of the respective premises. For the individual treatment rooms, the smaller manual diffusers Feel Happy (Primavera®) were used and filled with 4–6 drops of essential oils (for medium diffusion intensity, depending on the size of the respective treatment room, and per the intensity of diffusion in the waiting areas) and water (maximum capacity) three times a day, approximately every 3 h. The manual diffusers were cleaned with water daily before closing time. The app-controlled vaporizer was cleaned once a week on Friday just before closing time and filled with the essential oil for the following week. During the control week, all devices were switched off. The staff from all participating dental practices was instructed and trained by one member of the research team (JC) on the safe use of the equipment and the essential oils. In addition, the staff received detailed manuals on how to use the devices. The research team could be contacted at any time if questions arose.

### Sample size calculation

The required number of patients was calculated prospectively on the following basis: with a large effect size (0.7) (based on the published study populations of Lehrner et al., Zabirunnisa et al.^9,34^), alpha* = 0.013 (three patient groups), beta = 0.20 (power = 80%), ICC = 0.01, 47 patients* should be included per patient group and condition (essential oil and control groups), i.e., 705 patients* where planned in total (G*Power 3.1), i.e. 750 allowing for a dropout of 6%.

### Sample description

Adult patients at the four dental practices between 18 and 65 years during the data collection period who were willing were eligible to participate in the study. Patient recruitment (distribution of study information, obtaining informed consent, distribution, and collection of questionnaires) was undertaken by the individual dental practice staff. A cover story was initially used to blind the patients from the true study objective of testing essential oil vaporization effects on state anxiety and the perception of pain. The cover story described the subject of the study as an investigation of the effect of anxiety on pain perception, as was similarly done in a study by Lehrner et al.^33^.

Patients’ reasons for the visit to the dentist were clustered into three groups, “routine examination”, “acute pain” and “planned intervention”, since it can be assumed that the severity of anxiety also might depend on the planned treatment. Sociodemographic data was collected on the age range (18–30 years, 31–45, or 46–65) and gender (diverse, female, male).

### Data collection

The primary outcome measure for the study was the acute anxiety of the dental patients. In addition, three secondary outcomes were collected on trait anxiety, dental anxiety, and subjective pain perception during treatment. Acute and trait anxiety were measured with the state-trait-anxiety inventory (STAI). The STAI is a questionnaire that separately assesses state anxiety (= acute anxiety, STAI-S) and trait anxiety (= general disposition to anxiety, STAI-T) with 20 separate items using the most current, validated, and widely distributed version published in 1983 by Charles Spielberger. The German version used in the study AROMA_dent was developed and validated in 1981^46^. Participants were grouped by their score according to the trait anxiety scale with respect to a cutoff-score of ≤ vs > 42 as an average between the published cutoff-scores for STAI of 40 and 44^47–49^.

The second secondary outcome, dental anxiety, was collected with the Kleinknecht’s dental fear survey (KDFS)^50^. The questionnaire was translated into a German version. The translation process took place in accordance with relevant guidelines^51^.

The third and final secondary outcome of the patient’s subjective pain perception during treatment was collected by a numeric rating scale (NRS) ranging from 0 (= no pain) to 10 (= strongest pain imaginable).

The data was collected at two time points: the measurement of the data on anxiety by means of STAI and KDFS took place directly after the registration of the patients at the reception, allowing exposure to the essential oils for a short time (assumed 10–20 min) from the time they entered the practice until they completed the questionnaires, with documentation of acute anxiety with the STAI-S planned at the end of the questionnaire. The subjective pain perception of the patients was collected directly after the treatment by the dentist.

### Data analysis

The data was analyzed separately for each of the reason for visit patient groups, i.e., for patients visiting the practice for acute pain, for control or for planned procedures. For the primary outcome, acute anxiety prior to dental treatment was measured and compared between the four essential-oil conditions and the control condition. Independent ANCOVAs were calculated to estimate the contribution of the confounders: age, gender, trait anxiety and practice. For the secondary outcomes, the comparison between control condition and essential oil treatments was repeated regarding the outcome pain, and STAI-S was compared between blended essential oils vs. mono essential oils as well as forest-associated essential oils (Swiss Pine, Forest Walk) vs. fruit-associated essential-oils (Orange, Good Mood).

Only the three tests (for the three patient groups) for the primary endpoint were tested in confirmatory fashion using an adjusted alpha * of 0.0167. All of the other tests were assessed on an exploratory level only (against an unadjusted alpha = 0.05). Data was analyzed using custom-written python (version 3.9) routines and the statistical packages Statsmodels and Pingouin.

### Safety and adverse events

Since the patients were partially blinded by the cover story and the actual intervention (= essential oil vaporization in the dental practices) was undisclosed, the staff was asked to forward complaints expressed in relation to the odor in the dental practice to the research team.

### Ethics approval

The study was conducted according to the guidelines of the Declaration of Helsinki and approved by the Ethics Committee of Charité—Universitätsmedizin Berlin on August 20th, 2021 (EA2/197/21).

## Results

**Figure 2:**
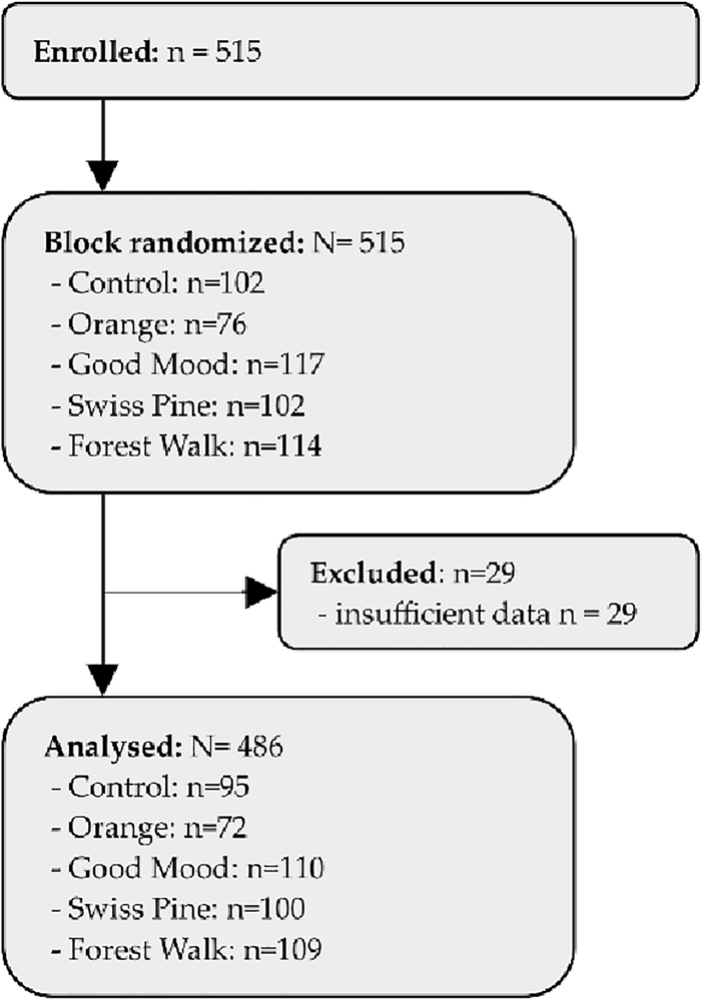
Consort flowchart.

515 patients of the originally intended 750 patients (thus 69%) could be included into the study. Of these, 486 patients (n = 296 females, n = 185 males, n = 5 diverse) completed at least the primary endpoint and the demographic data. The reason for the visit was marked as 44% for a routine examination, 44% for a planned intervention and the remaining 12% because of acute pain (see Fig. 2 and Table 2). As only 486 patients of the 705 planned patients were engaged, the assessed power is reduced from the usual 80 to 60.4%, thus reducing the probability to obtain significant results here (Table 3). We therefore showed effect sizes (Cohen’s d) next to the p-values in all tables.

**Table 2:** Baseline characteristics of the participants.

	Patients		Low STAI-T		High STAI-T	
Value	n	%	n	%	n	%
All patients	486	100	354	100	131	100
Reasons for treatment						
Planned	216	44.4	163	46	52	39.7
Acute	213	43.8	150	42.4	63	48.1
Routine	57	11.7	41	11.6	16	12.2
Gender						
Diverse	5	1	3	0.8	2	1.5
Female	296	60.9	200	56.5	95	72.5
Male	185	38.1	151	42.7	34	26
Age range						
18–30 years	94	19.3	70	19.8	24	18.3
31–45 years	194	39.9	143	40.4	50	38.2
> 46 years	198	40.7	141	39.8	57	43.5
Practice						
1	199	40.9	150	42.4	49	37.4
2	93	19.1	74	20.9	19	14.5
3	165	34	114	32.2	50	38.2
4	29	6	16	4.5	13	9.9
Treatment						
Control	95	19.5	67	18.9	28	21.4
Orange	72	14.8	54	15.3	18	13.7
Good Mood	110	22.6	80	22.6	29	22.1
Swiss Pine	100	20.6	72	20.3	28	21.4
Forest Walk	109	22.4	81	22.9	28	21.4

*n* count, *%* percentage with respect to the group of all patients, those with lower and higher trait anxiety.

**Table 3:** Results.

Parameter	Patients	Control			Intervention			t-test		
		n	*M*	*SD*	n	*M*	*SD*	*T*	*p*	d
Primary analysis										
STAI-T										
STAI-T	Low trait anxiety	67	35.9	7.68	287	35.3	8.94	0.60	0.552	0.07
	High trait anxiety	28	52.0	11.74	103	47.1	9.80	2.04	0.048	0.48
STAI-S										
STAI-S	All patients	95	40.7	11.64	391	38.4	10.54	1.76	0.080	0.21
	Reason for treatment									
	Routine	51	40.4	10.49	162	39.7	10.79	0.43	0.662	0.07
	Planned	9	47.6	15.29	48	41.6	10.53	1.12	0.291	0.53
	Acute	35	39.3	11.96	181	36.3	9.97	1.37	0.184	0.29
	Gender									
	Female	50	42.9	12.32	246	39.1	11.16	2.06	0.044	0.34
	Male	44	37.3	8.75	141	37.1	9.35	0.09	0.932	0.02
	Diverse	1	76	–	4	38.5	9.81	–	–	–
Kleinknecht	Dental fear									
	Avoidance	92	1.6	0.94	381	1.5	0.84	0.84	0.402	0.10
	Arousal	92	2.3	1.31	381	2.1	0.82	1.85	0.071	0.28
	Fear	92	2.5	1	378	2.2	0.92	1.98	0.052	0.24
Pain										
Pain NRS	All patients	91	1.6	1.63	379	1.3	1.69	1.31	0.193	0.15

Forest-associated essential oils vs. fruit-associated essential oil										
STAI-S	All patients	Swiss Pine + Forest Walk			Orange + Good Mood			t-test		
		209	38.8	10.29	182	37.8	10.84	0.97	0.331	0.10

Mono essential oils vs. blended essential oils										
STAI-S	All patients	Mono Oils: Orange + Swiss Pine			Blended Oils: Forest Walk + Good Mood			t-test		
		172	39.3	10.72	219	37.6	10.38	1.51	0.130	0.15
Additional analysis										
STAI-S	Reason for treatment in subgroup with high STAI-T									
	Routine	10	51.4	12.62	42	46.9	9.50	1.05	0.314	0.44
	Planned	15	50.1	10.21	48	46.4	10.33	1.22	0.235	0.36
	Acute	3	63.7	13.43	13	50.0	8.86	1.68	0.214	1.41
	Gender in subgroup with high STAI-T									
	Female	18	52.5	11.89	77	48.0	9.93	1.49	0.150	0.44
	Male	9	48.3	8.77	25	43.9	9.01	1.28	0.220	0.49
	Diverse	1	76.0	-	1	53.0	-	-	-	-
Kleinknecht	Dental fear in subgroup with high STAI-T									
	Avoidance	26	1.5	0.73	100	1.6	0.93	0.67	0.506	0.13
	Arousal	26	2.8	2.00	100	2.3	0.91	1.31	0.200	0.44
	Fear	26	2.8	0.99	100	2.6	1.03	0.89	0.377	0.19
Pain in subgroup with high STAI-T										
Pain NRS	High trait anxiety	25	1.5	1.78	101	1.3	1.48	0.73	0.468	0.18

Forest-associated essential oils vs. fruit-associated essential oil										
STAI-S	High trait anxiety	Swiss Pine + Forest Walk			Orange + Good Mood			t-test		
		56	46.7	9.81	47	47.5	9.87	0.39	0.698	0.08

Mono essential oils vs. blended essential oils										
STAI-S	High trait anxiety	Mono oils: Orange + Swiss Pine			Blended oils: Forest Walk + Good Mood			t-test		
		46	48.1	9.29	57	46.2	10.19	0.99	0.324	0.19

*n* count, *M* mean, *SD* standard deviation, *T and p* test statistic and resulting probability of the *t*-test, *d* effect size Cohen’s d.

### Primary analysis

**Figure 3:**
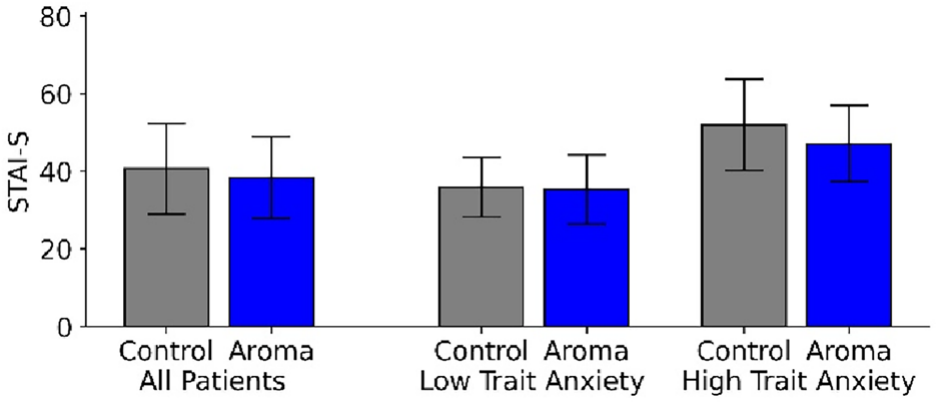
STAI-Acute results for all patients (left) and separated for patients with low trait anxiety (center) and high trait anxiety (right) for the control group (gray) and the Aroma groups (blue).

Figure 3 shows the results for the primary endpoint measure. Across all patients (n = 486), acute anxiety (STAI-S) was only marginally higher in the control group (*M* = 40.7, *SD* = 11.64) than in the groups treated with essential oils (*M* = 38.4, *SD* = 10.54), but this difference did not become significant, for any of three combined appointment reasons (*T* = 1.76, *p* = 0.080), nor for separate patient groups with routine examinations (*T* = 1.37, *p* = 0.184), planned examination (*T* = 0.43, *p* = 0.662) or acute pain (*T* = 1.12, *p* = 0.291). The huge standard deviations obtained here (amounting to more than a quarter of the mean value) indicate that inter-individual differences in state anxiety are rather large. Thus, we employed both pre-planned and additional post-hoc tests to explore whether any sub-groups of people could be identified by age, gender, treatment reason, trait anxiety and other confounders.

### Additional analysis

Additional ANCOVAs (run for patients with any reason together) revealed that neither the practices (*F* = 3.50, *p* = 0.062) nor age (*F* = 0.12, *p* = 0.772) showed any positive individual contribution (for these variables). In the post-hoc subgroup analysis of all female patients together (n = 296), state anxiety was significantly lower in the groups treated with essential oils compared to the control group (*p* = 0.044).

In an additional post-hoc analysis, the contribution of the trait anxiety was tested using a 2-dimensional ANOVA with “low vs. high STAI trait anxiety” as a second factor. Results revealed that trait anxiety is indeed a strong factor (*F(*1,482) = 184.7, *p* < 0.001). While for patients with a low trait anxiety (STAI-T score < 42), state anxiety were similar between the control (*M* = 35.9, *SD* = 7.68) and experimental group (*M* = 35.3, *SD* = 8.94), stronger differences emerged between the two groups for patients with high trait anxiety, i.e. with STAI-T scores ≥ 42 (*M* = 52.0, *SD* = 11.74 vs. *M* = 47.1, *SD* = 9.80) (right part of Figure 3).

**Figure 4:**
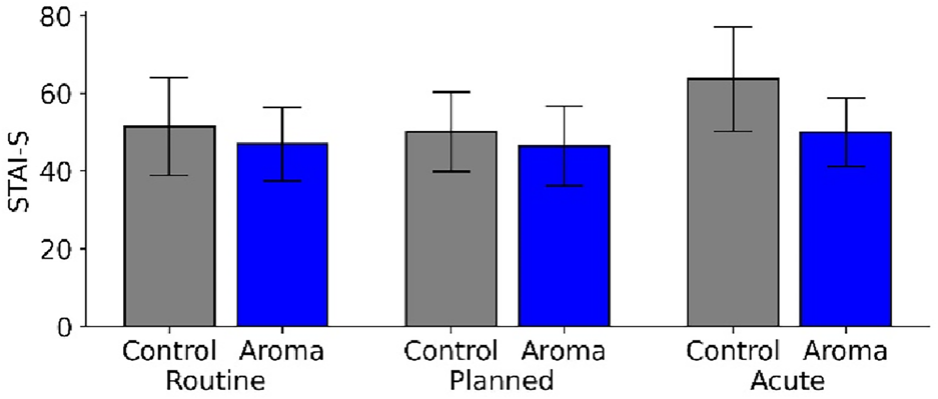
STAI-S results for patients with a higher STAI trait anxiety that underwent routine examination, planned interventions and acute pain treatment, respectively.

As a result, we confined all secondary analyses to the subpopulation with an increased level of trait anxiety (n = 131 patients): For this stratified sample, the difference in acute anxiety between the control groups (*M* = 52.0, *SD* = 11.74, n = 28) and the groups treated with essential oils (*M* = 47.1, *SD* = 9.80, n = 103) became significant (*T* = 2.04, *p* = 0.048), albeit with a non-clinically relevant difference of 4.9 (compared to the difference of 10 listed for the STAI-S as a minimal clinically important value^52^. However, possible confounders like the reason for the appointment (interaction: *F*(2,125) = 0.38, *p* = 0.986) (Fig. 4), gender (*F*(2,125) = 0.84, *p* = 0.434) or age group (*F*(2,125) = 2.65, *p* = 0.742) did not contribute significantly to the ANOVA model.

However, no significant differences were obtained for the three subscales of the Kleinknecht’s dental fear survey. The same holds true for the subjective pain perception of the subgroup in the intervention groups rated on an 11-point pain NRS. Data also revealed no significant differences for the other two secondary hypotheses. Patients treated with mono oils were minimally more anxious than patients treated with blended essential oils (*M* = 48.1, *SD* = 9.29 vs. *M* = 46.2, *SD* = 10.19, *T* = 0.99, *p* = 0.324). The comparison between forest-associated and orange-associated essential oils showed marginal differences that did not become significant (*T* = 0.39, *p* = 0.698). Anxiety levels in the groups with forest-associated scents were only slightly lower (*M* = 46.7, *SD* = 9.81) than in the groups with orange-associated essential oils (*M* = 47.5, *SD* = 9.87). Posthoc analyses further indicated that a possibly higher impact of the aromatherapy could be determined on the state anxiety in women (aromatherapy: *M* = 48.0, *SD* = 9.93 vs. control: *M* = 52.5, *SD* = 11.89) then in men, although the difference did not become significant (*T* = 2.79, *p* = 0.099) in the subgroup of female patients with a higher trait anxiety (n = 95), but for all female patients taken together (n = 296).

No adverse events and safety risks due to the essential-oil vaporization were reported by the dental practice staff.

To provide insights into (a) patients’ reasons for declining to participate in the study and (b) subjective perceptions on the essential-oil vaporization performed, anecdotal statements from dental office staff have been included (cf. Table 4). The potential of essential-oil vaporization that are not visible in the results of the questionnaires are highlighted in some of the anecdotal statements.

**Table 4:** Anecdotal statements from the employees about the intervention.

Topic	Reasons	Anecdotic statements from the staff
Refusal of the questionnaire by the patients	(1) Time-related (2) Questionnaire-related (3) Anxiety-related	(1) Waiting period between arrival and treatment was too short (time pressure) (2) Questionnaire was perceived as too long by the patients, fear of too much effort; language barrier existed (3) Some patients with high acute dental anxiety were unable to concentrate on the questionnaire; some patients refused to address the issue of anxiety, fearing that it would exacerbate their acute dental fear symptoms
Perceptions concerning the effects of essential-oil vaporization	(1) Forest Walk/Swiss Pine (2) Good Mood/Orange (3) Essential-oil vaporization in general	(1) Some employees found the scent too intense; one person associated the forest associated essential oils with causing headaches; some employees found the forest scents very pleasant and found they provided a good atmosphere in the practice (2) Many employees found the citrus scents relaxing and conducive to a beneficial atmosphere in the practice (3) Staff gave overall positive feedback on the essential-oil vaporization to provide a good, relaxing atmosphere

## Discussion

The main hypothesis that vaporization of the stress-reducing essential oils has a significant alleviating effect on acute anxiety in dental patients, compared to the control group, was suggested for the subgroup (n = 131) of all included patients with an increased level of trait anxiety. Although the difference of 4.9 points in state anxiety shown in the subgroup data does not correspond to an officially clinical relevant value^52^, it can be assumed that even modest statistical improvements in anxiety scores might have a high subjective value for those who are severely affected by acute anxiety in dental health contexts. Further RCTs are recommended to test the anxiolytic effects of essential oils in this specific subgroup with larger sample sizes. A mixed methods approach including qualitative research to explore subjective perceptions of essential oil applications would be appropriate to provide a more holistic understanding of this research field. Furthermore, in the subgroup of female participants (n = 296), the results show a distinct alleviating effect on acute anxiety. For the overall sample (n = 486), however, the effect was not proven to be statistically significant. This result might also be caused by large inter-individual differences (large SDs). Accordingly, post-hoc analysis indicated that this may be due to large differences between the subgroups defined by gender, and—even stronger—the degree of trait anxiety inherent in the patients.

This is consistent with other studies about aromatherapy and anxiety, which have shown little and non-significant improvement in anxiety symptoms for participants with mild anxiety, whereas participants with higher anxiety levels have demonstrated a stronger reaction to essential oil applications^12^. The anxiety-relieving potential of essential oils, specifically against DFA, has already been demonstrated in other empirical studies, however, within different designs and essential oils, particularly lavender^9,53–56^, and orange^33,34,54^. Our data also suggests that female dental patients may be more responsive than male patients to the effects of essential oils, a finding that has already been obtained elsewhere^33^. The stronger reaction of female patients to the vaporization of essential oils corresponds to the common assumption that women have better olfactory performance than men. A meta-analysis on “Sex Differences in Human Olfaction”^57^ confirms this assumption, differentiated in olfactory identification, threshold, and discrimination, albeit with a low effect size. There is no clear evidence of the reasons for this phenomenon, but hormonal, social, and cognitive differences are assumed (ibid.). However, the latter two differences appear to be due to gender rather than to sex. This raises the question of why the anxiety-reducing effect of essential oil vaporization in this study is higher in all women (n = 296), regardless of their level of trait anxiety, but not in the subgroup of women with high trait anxiety (n = 95). Although the difference in the subgroup did not become significant (*T* = 2.79, *p* = 0.099), we regard the tendency as important enough to consider it in future investigations.

Our data revealed that patients treated with mono oils were only minimally more anxious than patients treated with oil blends, thus, not statistically confirming the second hypothesis that presumed synergistic effects of blended essential oils compared to mono oils. Furthermore, the results do not support the hypothesis of significant pain relief from essential oil vaporization. Our hypothesis that nature-associated forest scents would have a greater effect than the other scents was also not confirmed with statistical significance. However, the forest-associated essential oil (blend) showed a tendency to slightly lower anxiety levels than the orange-associated essential oil (blend). The attempt to include culturally based odor preferences in the design to create a similar psychological effect (relaxation, well-being) in the most possible number of participants proved difficult to implement. This could be due to the inability to gather individual odor preferences for each participant in advance. Given the psychological effects of essential oils that are strongly related to the subjective olfactory evaluation, reactions to scent are individually dependent and culturally shaped by memories, experiences, and associated expectations. Therefore, the effects may be highly individual, and the same essential oil might trigger completely different reactions in different people^22,23^. Incorporating subjective preferences into a study design with a larger sample size is a major challenge. This is particularly true when the intervention involves vaporization of essential oils in large (hospital or practice) rooms and thus reaching many participants at the same time with the same essential oil application. The challenge to include subjective scent preferences under such circumstances should be addressed in future research. A conceivable approach could be to use fragrance dispensers that provide a selection of anxiety-relieving, calming essential oils to choose from.

A significant limitation within the study stemmed from dental practices as a research setting: due to the SARS-CoV-19 pandemic, there were significant staff shortages in all dental offices during the data collection period. The increased workload limited the ability of staff to include more patients in the study. In addition, the pandemic and associated restrictions reduced the number of patient visits. Thus, despite an extended data collection period in two of the dental practices (practice 1, 3), we were only able to recruit 69% of the intended sample size, reducing the power from the usual 80 to 60.4%.

A lack of demonstrated significance could be due to a number of reasons and limitations. Conditions in real-life settings do not allow for all variables to be (easily) controlled and predicted. For instance, evaporation intensity may have differentiated as adjustments were made by staff and the premises differed in terms of air circulations, number of rooms, size of the rooms, etc. The duration of individuals' exposure to the intervention in the waiting room during data collection, coupled with the effects of mandatory FFP2 mask-wearing, presents a challenge in terms of predictability. The estimated exposure time to the essential oils until data collection, ranging from 10 to 20 min, may have been relatively brief. Moreover, the mandatory use of masks (FFP2) in the practices during the survey period implies that both the duration and intensity of the intervention might have been constrained, potentially limiting its effects. It can be inferred that even modest reductions in dental anxiety, though substantial subjectively, may not be fully captured by statistical methods alone. Thus, for future research in this area, it is recommended to adopt an integrated mixed-method approach. This approach, ideally incorporating qualitative data alongside biosignal analysis, would provide a more nuanced and comprehensive understanding of the phenomenon.

In addition, collecting empirical data on essential-oil applications presents its own unique challenges^17,25,58^. An effect occurs not only because of the pharmacological mechanism of an essential oil, but also because of the psychological mechanisms that relate to subjective odor experiences, preferences and aversions^22,23^. Despite attempts to integrate individual psychological responses to smell by assuming a general association of forest scent with positive associations and relaxation, this approach appears inadequate given the results. The design did not allow for consideration of each participant's unique odor preferences. Consequently, due to the propensity of individual psychological responses to scents, essential oils may have triggered negative rather than stress-reducing responses in some patients based on their personal experiences. Considering that the characteristic smell in dental clinics is assumed one of the main factors for DFA^59^ and may serve as a trigger for acute anxiety, the scent of essential oils alone may have potential to reduce anxiety simply by masking the smells associated with the dental practice. However, the research design did not distinguish between the effects of essential oils and masked dental practice odors (e.g., by chemical fragrances).

Nevertheless, the results confirm the general, safe potential of essential-oil vaporization to alleviate high levels of dental anxiety. Consistent with several other studies on aromatherapy against anxiety, no adverse events were reported^12^. Moreover, anecdotal statements from dental practice staff (Table 4) suggest additional potential for enhancing the atmosphere and mood in the practice. This possibility should be investigated in future research and could be integrated as one part of a multimodal and holistic concept to address dental anxiety. This is bolstered by existing research, recommending holistic approaches and a combination of strategies to alleviate dental anxiety^5^.

A sustainable implementation of essential-oil vaporization in dental practices, for example as part of a comprehensive concept, could potentially yield benefits beyond the target group of patients. Anecdotal evidence from dental staff supports this assumption, suggesting that essential oil vaporization might have an impact not only patients with dental anxiety but also on the dental staff. The stress-reducing potential of essential oils may influence the entire practice environment, extending its effects beyond the intended patient group. If improved atmosphere and mood in the practice were to result, it could potentially create a positive space and environment conducive to a reducing in patient stress and anxiety. This assumption aligns with the growing research on healing architecture^60–63^. Further research on the importance of a healing environment in dental spaces and the potential of essential-oil vaporization in this context is required.

In conclusion, these results add to the large body of evidence from research on anxiety-relieving effects of essential oil vaporization from the perspective of dental practices. Despite the heterogenous nature of studies, effective use of essential oils has been demonstrated to some extent. The real potential, however, seems to lie in the complex psychological, individual responses to scent, which can invoke a large range of emotions and memories that may influence mood, including anxiety.

## Conclusion

Across all patients (n = 486), acute anxiety (STAI-S) was marginally higher in the control group than in the groups treated with essential oils, but this difference showed not to be significant. The same applies to the other hypotheses: essential oil blends were not shown to have a stronger anxiety-relieving effect compared to singular compound oils; forest-associated essential oils showed only a slightly stronger anxiety-relieving effect than the citrus-associated oils and no effect on pain perception during treatment was observed.

However, the results of the study confirm the potential of essential-oil vaporization to alleviate acute anxiety in the subgroup of patients with a high level of trait anxiety (n = 131) and in the subgroup of female patients (n = 296). Furthermore, the stress-reducing potential of the essential-oil vaporization was confirmed by the anecdotic statements of dental-practice staff who noted a positive effect on the atmosphere and mood in the practice. Based on these promising findings, the favorable cost-effectiveness and the safe and easy application of essential oil vaporization compared to the administration of pharmaceuticals, the use of aromatherapy in dental practices is recommended for anxiety reducing strategies. Further research should consider using multimethod approaches and including the dental office staff in the target population to obtain a more holistic picture of aromatherapy approaches in dental practices. In addition, the anxiety-reducing effect should be studied in a larger population of (a) female patients, and (b) patients with a high level of trait anxiety, not wearing FFP2 masks. Future research should also consider longitudinal studies for further insights into the sustainability of effects over time. Furthermore, investigating the role of essential-oil vaporization in reducing anxiety within a multifaceted intervention aimed at managing DFA could be of interest. Further exploring and harnessing the potential of aromatherapy as a strategy against DFA and to cultivate a relaxing atmosphere in dental practices presents an important avenue for future research.

## Data Availability

The datasets used and/or analysed during the current study are available from the corresponding author on reasonable request.

## Abbreviations

DFA: Dental fear and anxiety
STAI: State-trait-anxiety inventory
STAI-S: State version of the state-trait-anxiety inventory
STAI-T: Trait version of the state-trait-anxiety inventory
KDFS: Kleinknecht dental fear survey
NRS: Numeric rating scale
CIM: Complementary and integrative medicine
